# Development of an RNA Virus-Based Episomal Vector Capable of Switching Transgene Expression

**DOI:** 10.3389/fmicb.2019.02485

**Published:** 2019-11-06

**Authors:** Yusuke Yamamoto, Keizo Tomonaga, Tomoyuki Honda

**Affiliations:** ^1^Laboratory of RNA Viruses, Department of Virus Research, Institute for Frontier Life and Medical Sciences, Kyoto, Japan; ^2^Laboratory of RNA Viruses, Graduate School of Biostudies, Kyoto, Japan; ^3^Department of Molecular Virology, Graduate School of Medicine, Kyoto University, Kyoto, Japan; ^4^Division of Virology, Department of Microbiology and Immunology, Osaka University Graduate School of Medicine, Osaka, Japan

**Keywords:** Borna disease virus, virus vector, riboswitch, expression control, safety

## Abstract

Viral vectors are efficient gene delivery systems, although most of these vectors still present limitations to their practical use, such as achieving only transient transgene expression and a risk of insertional mutations. We have recently developed an RNA virus-based episomal vector (REVec), based on nuclear-replicating Borna disease virus (BoDV). REVec can transduce transgenes into various types of cells and stably express transgenes; however, an obstacle to the practical use of REVec is the lack of a mechanism to turn off transgene expression once REVec is transduced. Here, we developed a novel REVec system, REVec-L2b9, in which transgene expression can be switched on and off by using a theophylline-dependent self-cleaving riboswitch. Transgene expression from REVec-L2b9 was suppressed in the absence of theophylline and induced by theophylline administration. Conversely, transgene expression from REVec-L2b9 was switched off by removing theophylline. To our knowledge, REVec-L2b9 is the first nuclear-replicating RNA virus vector capable of switching transgene expression on and off as needed, which will expand the potential for gene therapies by increasing safety and usability.

## Introduction

Increasing lists of causative genes for genetic disorders and gene delivery systems have led to the development of gene therapies over several decades. In particular, the development of efficient and safe gene delivery systems is critical to introduce genes to treat genetic disorders. To date, various non-viral gene delivery systems, such as cationic liposome technology (Miller, 1998), and viral delivery systems, such as adeno virus-, adeno-associated virus (AAV)-, and lentivirus-based vectors, have been developed (Escors and Breckpot, 2010; Crystal, 2014; Naso et al., 2017). Although these systems can be used to successfully transduce a gene of interest, they still present disadvantages such as achieving only transient expression of transgenes, cytotoxicity, and the potential for integration of viral vector sequences into the host genome. Moreover, to maximize the effect of transgenes, transgene expression should be controlled at an appropriate level and with appropriate timing. Thus, possible requirements for an ideal gene delivery system include long-term expression of transgenes, minimal cytotoxicity, reduction of integration risk, and controllable expression of transgenes. However, none of the delivery systems described above meet all of these requirements at present.

Recently, oncolytic virus therapy has been increasingly appreciated as a promising and realistic approach for treating cancer. For example, herpes simplex virus type 1, vaccinia virus, adenovirus, and reovirus are major oncolytic viruses undergoing clinical trials (Hu et al., 2006; Ramesh et al., 2006; Vidal et al., 2006; Parato et al., 2012). To increase the oncolytic activity of these viral therapies, the viruses are often genetically engineered, such as through the insertion of human granulocyte macrophage colony-stimulating factor (GM-CSF) (Hu et al., 2006). Because these modifications may harm the host, the use of viral vectors, in which the expression of transgenes can be controlled, will be a safer therapeutic option.

In previous studies, we established a Borna disease virus (BoDV)-based vector, the RNA virus-based episomal vector (REVec) system (Daito et al., 2011). BoDV is a non-segmented, negative strand RNA virus that exhibits several unique biological characteristics. For example, BoDV replicates in the nucleus without overt cytopathic effects (Ludwig and Bode, 2000). Furthermore, the ribonucleoprotein complex (RNP) of BoDV interacts with the host chromosome (Matsumoto et al., 2012), which enables stable persistent infection in the nucleus. Although integrations of BoDV sequences to the host genome have been reported (Horie et al., 2010), the probability is extremely low (Horie et al., 2013). These characteristics indicate that BoDV could be an ideal RNA viral vector for safe, efficient, and long-term transgene expression. The REVec system expresses transgenes from an additional transcription cassette in the BoDV genome between the phosphoprotein (P) and matrix (M) genes (Daito et al., 2011). REVec carrying the *GFP* gene, REVec-GFP, achieved long-term expression of GFP in both cultured cells and mouse brains (Daito et al., 2011). We have also reported that REVec can express arbitrary miRNAs and silence their target genes (Honda et al., 2016). Moreover, REVec can be efficiently transduced in human pluripotent stem cells (iPSCs) without disturbing the pluripotency of the cells (Ikeda et al., 2016; Komatsu et al., 2019). Recently, we developed a REVec system lacking both the M and glycoprotein (G) genes, REVecΔMG (Fujino et al., 2017). REVecΔMG seems to be safer than the original REVec because REVecΔMG does not express the M and G proteins, which are required for viral particle production and transmission and may induce cytotoxicity (Cathomen et al., 1998; Bajramovic et al., 2003; Honda et al., 2009). Although the REVec system is a unique and safe RNA viral vector, one concern about the system is the lack of a mechanism for regulating transgene expression from REVec in transduced cells. Therefore, it is important to develop a version of REVec capable of controlling transgenes for practical use.

**FIGURE 1:**
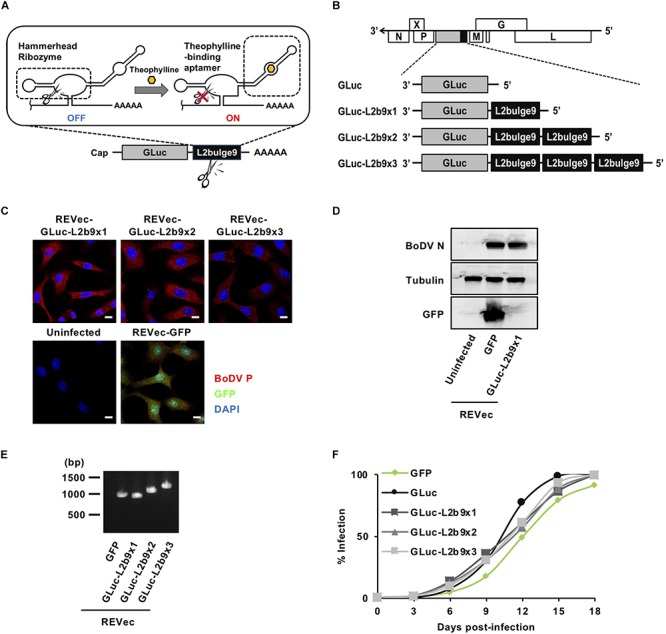
Generation of REVec capable of controlling transgene expression. **(A)** Schematic diagram of L2b9. **(B)** Representation of the REVec-Gluc-L2b9 vector genomes. **(C)** IFA of established REVec-L2b9-infected Vero cells. Bars, 10 μm. **(D)** N protein expression in established REVec-L2b9-infected cells. Lysates of REVec-L2b9-infected cells were subjected to western blotting using anti-N, anti-tubulin, and anti-GFP antibodies. **(E)** RT-PCR of the L2b9 cassette in REVec-L2b9. Copies of GLuc-L2b9 in each established REVec-L2b9 vector were detected by RT-PCR. **(F)** Growth kinetics of REVec-GFP, REVec-GLuc, and REVec-GLuc-L2b9s. Vero cells were *de novo* infected with REVecs at an MOI of 0.01, and the viral growth rate was monitored by IFA.

Riboswitches are ligand-binding RNA structures that serve as molecular switches (Serganov and Nudler, 2013). Riboswitches can modulate transcription, translation, splicing and mRNA stability in bacteria and eukaryotes. For example, in different groups of eukaryotes, thiamine pyrophosphate riboswitches regulate genes by alternative splicing in filamentous fungi (Cheah et al., 2007), regulate premature translation termination in green algae (Croft et al., 2007), and regulate splicing and mRNA degradation in higher plants (Bocobza et al., 2007; Wachter et al., 2007). Based on the natural riboswitch mechanism, various synthetic riboswitches have been developed, such as a tetracycline-dependent translational riboswitch in yeast (Berens et al., 2001; Suess et al., 2003). The *cis*-acting self-cleaving riboswitch L2bulge9 (L2b9) is an “ON” riboswitch consisting of a theophylline-dependent aptamer and a self-cleaving ribozyme (Figure 1A; Win and Smolke, 2007). When theophylline is unbound to the aptamer domain, the self-cleaving domain is in an active form, which cleaves itself and causes mRNA degradation. On the other hand, when theophylline binds to the aptamer domain, the ribozyme domain shifts to an inactive form, which blocks ribozyme cleavage, stabilizing mRNA and restoring mRNA translation. Since this riboswitch has been confirmed to function in mammalian cells, we reasoned that a novel REVec system capable of controlling transgene expression could be developed using the L2b9 riboswitch.

In this study, we generated a novel RNA viral vector, REVec-L2b9, capable of controlling transgene expression using a theophylline-dependent self-cleaving riboswitch. Transgene expression from REVec-L2b9 was successfully suppressed in the absence of theophylline and was induced by theophylline in a dose-dependent manner. Notably, removal of theophylline re-suppressed transgene expression, demonstrating that transgene expression from REVec-L2b9 can be controlled as needed.

## Materials and Methods

### Cells

Puromycin-resistant Vero cells (a monkey kidney cell line) were cultured in Dulbecco’s modified Eagle’s medium (DMEM) (Nacalai Tesque, Kyoto, Japan) supplemented with 2% fetal calf serum (FCS). 293T cells (a human embryonic kidney cell line) were cultured in DMEM supplemented with 10% FCS.

### Plasmids

The REVec plasmid harboring an extra transcription cassette, pBoDV, was generated as described previously (Daito et al., 2011). The self-cleaving riboswitch L2bulge9 (L2b9), described previously (Win and Smolke, 2007), was synthetized by PCR with the appropriate primers (Supplementary Table S1). The *Gaussia* Luciferase (GLuc) gene was subcloned upstream of L2b9 (GLuc-L2b9x1) and L2b9 tandem repeats (GLuc-L2b9x2 and GLuc-L2b9x3). pBoDV-GLuc-L2b9x1, pBoDV-GLuc-L2b9x2 and pBoDV-GLuc-L2b9x3 were generated by the insertion of GLuc-L2b9 sequences into the *Bst*BI and *Pac*I sites of pBoDV. The *Ras-related C3 botulinus toxin substrate 1* (*Rac1*, GenBank: AF498964.1) gene was cloned from the cDNA of 293T cells. The constitutively active form of Rac1 (RacQ61L) was generated by site-directed mutagenesis. pBoDV-RacQ61L-L2b9x2 was generated by the insertion of the RacQ61L sequence into the *Bst*BI and *Sbf1* sites of pBoDV-GLuc-L2b9x2.

### Reverse Genetics of REVec

Recombinant viruses were generated with reverse genetics technology established by our group (Daito et al., 2011). 293T cells were transfected with these BoDV-expressing plasmids and the helper plasmids expressing the BoDV N, P, and L genes using Lipofectamine^®^ 2000 (Thermo Fisher Scientific, Waltham, MA, United States). At 3 days post-transfection, the transfected 293T cells, which were REVec-producing cells, were passaged. One day after the first passage, puromycin-resistant Vero cells were overlaid and co-cultured with the REVec-producing 293T cells to propagate REVec by the cell-to-cell transmission between 293T and Vero cells. Then, the cells were passaged every 3 days in the presence of puromycin to remove the transfected 293T cells. After several weeks of the culture, we finally obtained REVec-infected Vero cells. The resulting REVecs were REVec-GFP, REVec-GLuc, REVec-GLuc-L2b9x1, REVec-GLuc-L2b9x2, REVec-GLuc-L2b9x3, REVec-RacQ61L, and REVec-RacQ61L-L2b9x2. The established REVec-infected Vero cells were evaluated for BoDV proteins by immunofluorescence assay (IFA) and western blotting to confirm REVec production and used for most of the experiments except for *de novo* infection experiments.

### Virus Preparation

REVec-infected Vero cells were collected and washed with phosphate-buffered saline (PBS). The cells were sonicated in DMEM supplemented with 2% FCS and then the samples were centrifuged to remove cell debris. After centrifugation, the supernatants were collected as virus stocks.

### *De novo* Virus Infection

Vero cells were infected with REVecs at a multiplicity of infection (MOI) of 0.01 at 37°C. After viral absorption for 1 h, the cells were washed with PBS and passaged every 3 days. Virus propagation was detected by IFA.

### IFA

Immunofluorescence assay was conducted as described previously (Kojima et al., 2014) with some modifications. Briefly, cells were fixed for 20 min in 4% paraformaldehyde and permeabilized by incubation in PBS containing 0.25% Triton X-100 for 10 min. After permeabilization, the cells were incubated with a rabbit anti-BoDV P antibody for 1 h. This was followed by incubation with the appropriate Alexa Fluor-conjugated secondary antibodies (Invitrogen, Carlsbad, CA, United States). The cells were counterstained with 4′,6-diamidino-2-phenylindole (DAPI). REVec-GLuc-L2b9x2-, REVec-RacQ61L-L2b9x2-, and REVec-RacQ61L-infected Vero cells were incubated with a rabbit anti-P antibody for 1 h. This was followed by incubation with Acti-stain 488 phalloidin (Cytoskeleton Inc., Denver, CO, United States), DAPI and the appropriate Alexa Fluor-conjugated secondary antibody for 1 h. An ECLIPSE Ti confocal laser-scanning microscope (Nikon, Shinagawa, Japan) was used for cell immunofluorescence imaging and data collection.

### Luciferase Assay

Theophylline (Wako, Osaka, Japan) was dissolved in DMEM supplemented with 2% FCS. REVec-GLuc-infected Vero cells (4 × 10^4^/well) were seeded into 48-well plates. At 3 h after seeding, the cells were treated with 0, 1, 3, or 10 mM theophylline. After 24 h of incubation, the luciferase activity in the culture medium was measured using a Lumat LB 9507 luminometer (Berthold, Bad WildBad, Germany) and a BioLux *Gaussia* Luciferase Assay Kit (New England Biolabs, Ipswich, MA, United States) according to the manufacturer’s instructions. The cells were counted with a hemocytometer, and luciferase activities were normalized by the cell counts.

### Western Blot Analysis

Vero cells infected with REVec were lysed with SDS sample buffer. The total cell lysate was subjected to SDS-PAGE and transferred to a PVDF membrane. The membranes were then blocked and incubated with the primary antibodies. The antibodies used in this study were as follows: mouse anti-BoDV N (HN132), mouse anti-GFP (Takara Bio Inc., Shiga, Japan) and mouse anti-Tubulin (Sigma-Aldrich, St. Louis, MO, United States) antibodies. After three washes with 0.05% Tween 20 in Tris-buffered saline (TBS), horseradish peroxidase-conjugated secondary antibodies (Invitrogen, Waltham, MA, United States) were applied for 1 h at room temperature. The bound antibodies were detected using an ECL Prime Western Blotting System (GE Healthcare Bioscience, Piscataway, NJ, United States) and a FUJIFILM LAS-4000 Mini Lumino Image analyzer (Fujifilm, Tokyo, Japan).

### RNA Preparation and Real-Time RT-PCR

Total RNA was extracted from the REVec-infected cells using TRIzol (Life Technologies, Grand Island, NY, United States) and reverse transcribed with a Verso cDNA Synthesis Kit (Thermo Fisher Scientific, Waltham, MA, United States) using the primers shown in Supplementary Table S1. qRT-PCR assays of REVec genomic and antigenomic RNA were carried out using a gene-specific double fluorescence-labeled probe and THUNDERBIRD Probe qPCR Mix or THUNDERBIRD SYBR qPCR Mix (Toyobo, Osaka, Japan) in a Rotor Gene Q 2plex HRM system (Qiagen, Hilden, Germany). The primers and probe used are shown in Supplementary Table S1.

## Results

### Generation of REVec Capable of Controlling the Expression of a Transgene

To develop a version of REVec capable of controlling transgene expression in vector-transduced cells, we generated pBoDV plasmids harboring one to three copies of L2b9, a theophylline-dependent self-cleaving riboswitch, in the 3′ UTR of the *Gaussia luciferase* (GLuc) gene, inserted between the P and M genes of the BoDV genome (Figure 1B). Using these plasmids, we generated REVecs (REVec-GLuc-L2b9x1, REVec-GLuc-L2b9x2, and REVec-GLuc-L2b9x3) via a reverse genetics approach established previously (Daito et al., 2011). We successfully obtained Vero cells infected with REVec-GLuc-L2b9x1, REVec-GLuc-L2b9x2, and REVec-GLuc-L2b9x3 (Figures 1C,D). We confirmed that the repeats of the L2b9 sequence were retained in the vector genome after establishing REVec-infected Vero cells (Figure 1E). Although REVec-GLuc-L2b9s contained the L2b9 ribozyme sequence in the REVec antigenomic RNAs, the replication kinetics of REVec-GLuc-L2b9s were comparable to those of REVec-GFP and REVec-GLuc during the *de novo* infection (Figure 1F). These results indicate that the insertion of the L2b9 sequence in REVec antigenomic RNA does not induce any deleterious effects on vector replication in the infected cells.

### Induction of Transgene Expression From REVec-GLuc-L2b9 by Theophylline

**FIGURE 2:**
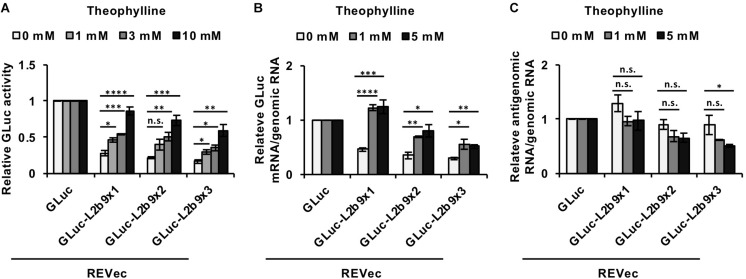
Induction of transgene expression from REVec-GLuc-L2b9 by theophylline. **(A)** Relative luciferase activity after 24 h of treatment with 0, 1, 3, and 10 mM theophylline. Luciferase activities were normalized by the cell counts. **(B,C)** Quantification of RNA expression in REVec-GLuc-L2b9-infected Vero cells by qRT-PCR. The amounts of GLuc mRNA **(B)** and REVec antigenomic RNA **(C)** were measured and standardized against that of REVec genomic RNA. Values are expressed as the mean ± S.E. ^∗^*P* < 0.05; ^∗∗^*P* < 0.01; ^∗∗∗^*P* < 0.005; ^****^P < 0.001 (Student’s *t*-test). At least three experiments were performed.

We then assessed whether the administration of theophylline could induce luciferase expression in cells infected with REVec-GLuc-L2b9s. As shown in Figure 2A, in the absence of theophylline, the luciferase activities derived from REVec-GLuc-L2b9-infected cells were reduced with an increase in the number of the L2b9 sequences, indicating that the L2b9 sequence suppresses luciferase expression. When theophylline was added to the cells, luciferase expression was induced in a dose-dependent manner (Figure 2A). To evaluate whether the increase in luciferase activity induced by theophylline in REVec-GLuc-L2b9-infected cells resulted from mRNA stabilization of GLuc, we evaluated the amount of GLuc mRNA in the presence or absence of theophylline. As shown in Figure 2B, the amount of GLuc mRNA per REVec genomic RNA was increased by the administration of theophylline. On the other hand, the amount of REVec antigenome RNA per REVec genomic RNA was unaffected at all tested theophylline concentrations (Figure 2C). These results demonstrate that theophylline administration can induce transgene expression from REVec-L2b9 by stabilizing transgene mRNAs, as expected.

### Reversible Control of Transgene Expression From REVec-GLuc-L2b9 by Theophylline

**FIGURE 3:**
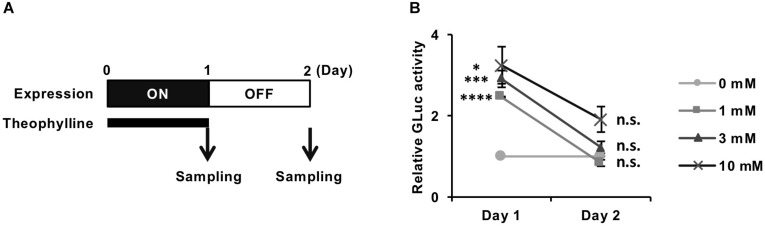
Reversible control of transgene expression from REVec-GLuc-L2b9 by theophylline. **(A)** Time course of switching off the expression from REVec-GLuc-L2b9. Theophylline treatment was ceased on Day 1. The culture media were collected at the indicated time points, and luciferase activity was measured. **(B)** Relative luciferase activity at Days 1 and 2. Theophylline treatment at the indicated concentration was ceased on Day 1. Luciferase activities were normalized by the cell counts. Values are expressed as the mean ± S.E. ^∗^*P* < 0.05; ^∗∗∗^*P* < 0.005; ^****^P < 0.001; n.s., no significance (Student’s *t*-test; vs. 0 mM). At least three experiments were performed.

We next evaluated whether the theophylline-induced luciferase expression from REVec-GLuc-L2b9 returns to the basal level after cessation of the treatment. We treated REVec-GLuc-L2b9-infected cells with theophylline for 1 day, subsequently washed the cells, and cultured the cells in the absence of theophylline for one additional day (Figure 3A). As shown in Figure 3B, luciferase activity was induced by theophylline treatment (Day 1) and returned to the basal level after the removal of theophylline (Day 2), demonstrating that transgene expression from REVec-L2b9 can be switched on and off by theophylline as needed.

### Control of Cell Morphology Using the REVec-L2b9 System

**FIGURE 4:**
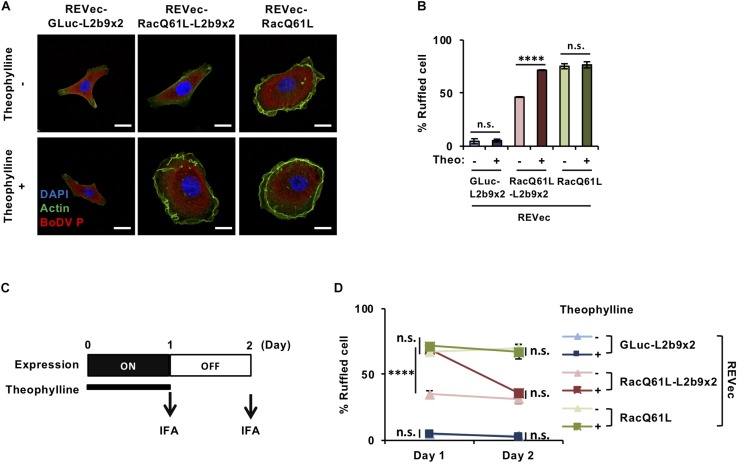
Control of cell morphology using the REVec-L2b9 system. **(A)** IFA of Vero cells infected with REVec-GLuc-L2b9x2, REVec-RacQ61L-L2b9x2, and REVec-RacQ61L. Cells were incubated with or without 3 mM theophylline for 1 day. Bars, 10 μm. **(B)** The percentages of ruffled cells. Cells were incubated with 3 mM theophylline for 1 day, and cell morphology was observed by IFA. **(C)** Time course of switching off the expression from REVec-RacQ61L-L2b9x2. Cells were incubated with 3 mM theophylline for 1 day, and then theophylline treatment was ceased on Day 1. **(D)** The percentages of ruffled cells on Days 1 and 2. Theophylline treatment was ceased on Day 1. Cell morphology was observed by IFA. Values are expressed as the mean ± S.E. ^****^P < 0.001; n.s., no significance (Student’s *t*-test). At least three experiments were performed.

Finally, we evaluated whether our system is effective enough to function in biological contexts. To this end, we tried to modulate cell morphology using the REVec-L2b9 system. Rac1 (Ras-related C3 botulinus toxin substrate 1) is a small GTPase that regulates various biological processes, including cell membrane ruffling (Hall, 1998). A constitutively active mutant of Rac1, RacQ61L, induces cell membrane ruffling and bubble-like structures (Zhang et al., 1999; Davis et al., 2013). To regulate cell membrane ruffling as needed, we inserted RacQ61L into the pBoDV-L2b9x2 plasmid, generated REVec-RacQ61L-L2b9x2 (Supplementary Figure S1), and established REVec-transduced Vero cells by reverse genetics. In the absence of theophylline, REVec-RacQ61L-transduced Vero cells exhibited marked membrane ruffling, whereas REVec-GLuc-L2b9x2- and REVec-RacQ61L-L2b9x2-transduced cells showed less ruffling (Figures 4A,B). When theophylline was added, membrane ruffling was induced in REVec-RacQ61L-L2b9x2-transduced cells, while that in REVec-RacQ61L- and REVec-GLuc-L2b9x2-transduced cells was not affected (Figures 4A,B). These results indicate that the expression of RacQ61L from REVec-RacQ61L-L2b9x2 is induced by theophylline administration, which triggers membrane ruffling in the transduced cells. Again, the levels of REVec antigenome RNAs were unchanged by theophylline administration (Supplementary Figure S2), confirming that this control is specific to the expression of the L2b9-containing transgene from REVec. We then evaluated whether theophylline-induced membrane ruffling disappears after the cessation of theophylline treatment. To this end, we treated REVec-transduced Vero cells with theophylline for 1 day and then ceased the treatment, as shown in Figure 4C. The theophylline-induced membrane ruffling of REVec-RacQ61L-L2b9x2-transduced cells decreased within 1 day after the cessation of treatment (Figure 4D). On the other hand, the membrane ruffling of REVec-RacQ61L- and REVec-GLuc-L2b9x2-transduced cells was comparable regardless of the presence of theophylline (Figure 4D). These results demonstrate that the REVec-L2b9 system is effective enough to modulate a biological process.

## Discussion

Viral vector systems are widely used not only in basic researches but also in gene and cellular therapies. Although viral vectors based on lentiviruses/retroviruses, adenoviruses and AAVs are commonly chosen in clinical studies and applications at present, these vectors still present some disadvantages. For instance, adenovirus vectors induce cytopathic effects and immune responses (Franklin et al., 1999; Muruve, 2004). Integrating viral vectors, such as lentivirus vectors, cause insertional mutagenesis and oncogenesis (Schambach et al., 2013). Transgene expression from AAV vector is transient because of the loss of AAV vector genomes in dividing tissues (Colella et al., 2018). On the other hand, RNA virus vectors, including vesicular stomatitis virus (VSV) and Sendai virus vectors, are safer than lentivirus and DNA virus vectors since they present a lower risk of viral genome integration into the host genome. However, most RNA virus vectors cannot transduce transgenes for a long period of time due to virus extinction, which is a weakness of these vectors.

REVec is unique among RNA virus vectors because it can establish long-lasting persistent infection in the nucleus as an episomal RNA associated with the cellular chromosomes, supporting long-term expression of transgenes. Therefore, REVec is an ideal virus vector for safe and long-term gene delivery. Although REVec is ideal for long-term gene delivery, sustained transgene expression cannot be turned off once REVec is transduced. T-705 is a candidate chemical that can eliminate REVec from the transduced cells in the case of adverse reactions from sustained transgene expression (Tokunaga et al., 2017). However, if T-705 eliminates REVec from the transduced cells in such cases, re-transduction of the vector is required for the re-expression of transgenes. To further increase the safety and usefulness of REVec, a vector capable of controlling transgene expression would be ideal. Additionally, the activation and silencing of therapeutic transgenes will be critical to moderate recurrent symptoms of chronic diseases, such as epilepsy (Weinberg and McCown, 2013), in a timely manner and to avoid side effects due to sustained overexpression of transgenes. Viral vectors in which a transgene is repressed by constant administration of substances, such as antibiotics, may cause potential clinical problems. Therefore, the use of transgene-inducible systems that can reversibly induce transient transgene expression may be the best option for obtaining a safer and useful therapeutic tool.

Among the existing gene regulatory systems, the tetracycline (Tet)-dependent transcriptional switch is the most widely exploited system for controlling transgene expression. The Tet regulatory system has been encoded within AAVs (McGee Sanftner et al., 2001), high-capacity helper-dependent adenoviruses (Salucci et al., 2002), lentiviruses (Kafri et al., 2000; Johansen et al., 2002; Régulier et al., 2002) and retroviruses (Iida et al., 1996), achieving successful gene regulation. However, because non-retroviral RNA viruses, including REVec, replicate without a known DNA intermediate stage, transcriptional regulation using Tet is not applicable to the RNA virus vector systems. To regulate transgene expression from REVec, we therefore sought to apply an RNA-based regulatory system: a riboswitch.

Although a self-cleaving riboswitch in the genomic or antigenomic RNA of RNA viruses seems to be deleterious to their replication, several studies have succeeded the application of a self-cleaving riboswitch to the engineering of cytoplasmic-replicating RNA viruses thus far (Ketzer et al., 2014; Bell et al., 2015; Takahashi and Yokobayashi, 2019). In the case of a vector based on an alphavirus (a positive-stranded RNA virus) with a theophylline-dependent self-cleaving riboswitch, theophylline is required for stabilizing the virus genome and generating the vector (Bell et al., 2015), consistent with our concern. Because transgene mRNA synthesis coincides with alphavirus replicon replication, switching off transgene expression results in the extinction of the vector. In contrast, we successfully rescued REVec-L2b9 regardless of theophylline administration. Furthermore, the induction of transgene expression from REVec-L2b9 by theophylline was shown to be reversible (Figures 2A, 3B, 4B,D), while the amount of the vector was not affected by theophylline (Figure 2C and Supplementary Figure S2). This may have occurred because, unlike alphavirus vector, which contains a positive-strand RNA genome, newly synthesized REVec antigenomic RNA molecules are co-transcriptionally packaged by the N protein into RNP (Honda and Tomonaga, 2013), which prevents the antigenomic RNA from undergoing self-cleavage by the riboswitch.

REVec-L2b9 has several advantages over the reported cytoplasmic-replicating RNA virus vector systems using riboswitches. Among the reported systems (Ketzer et al., 2014; Bell et al., 2015; Takahashi and Yokobayashi, 2019), only REVec and VSV-based vectors have been evaluated to control the transgene expression by riboswitches without affecting the vector replication. REVec-L2b9 induced the transgene expression within 24 h (Figure 2A), while the VSV-based systems took 48 h (Takahashi and Yokobayashi, 2019). This suggests that the transgene induction from REVec-L2b9 might be faster than that from the VSV-based systems. Additionally, REVec has an advantage in long-term transduction of transgenes, since the transgene expression of the VSV-based vectors is generally transient, while REVec has been reported to express the transgene for at least 8 months (Daito et al., 2011). Furthermore, we demonstrated for the first time that REVec-L2b9 could control a biological process (Figure 4), whereas only regulation of reporter gene expressions was evaluated in the VSV-based systems (Takahashi and Yokobayashi, 2019).

The ratio of transgene expression in the “ON” state to the “OFF” state obtained in this study might not be high enough for the practical use of REVec-L2b9. To solve this problem, we sought to increase the number of the L2b9 sequences in the transgene mRNA. With the increase in the L2b9 sequences, the amount of transgene expression in the “OFF” state was decreased as expected (Figure 2A and Supplementary Figure S3A). This result indicates that REVec carrying more L2b9 sequences reduces the risk of unexpected consequences caused by sustained transgene expression. In contrast to the “OFF” state, transgene expression in the “ON” state was comparable regardless of the number of riboswitches (Supplementary Figure S3B). Improvement of transgene silencing in the “OFF” state and transgene expression in the “ON” state by optimizing the aptamer-ribozyme and aptamer-compound sets will further increase the safety and usefulness of the REVec system.

In conclusion, to our knowledge, this is the first study to develop a nuclear-replicating RNA virus vector whose transgene expression can be switched on and off at any time point as needed. REVec-L2b9 is a safe and useful system for gene delivery, although some improvements can be introduced for future practical applications. For example, we used transmission-competent REVec as a platform for this proof-of-concept study because of easy preparation and handling. However, it is expected to be safer if we introduce the L2b9 riboswitch to transmission-defective REVecΔMG (Fujino et al., 2017). Additionally, REVec still presents some disadvantages: i.e., it takes a long time to generate recombinant viral vectors and the vector yield is not high. By improving the efficiency of vector preparation and transgene expression control, REVec-L2b9 will become a remarkable option for safer gene and cellular therapies with a fail-safe switch for transgene expression *in vivo*.

## Data Availability Statement

The datasets generated for this study are available on request to the corresponding author.

## Author Contributions

YY and TH conducted the experiments and analyzed the data. KT and TH conceived and designed the study. YY, KT, and TH wrote the manuscript.

## Conflict of Interest

The authors declare that the research was conducted in the absence of any commercial or financial relationships that could be construed as a potential conflict of interest. The reviewer NP declared a past collaboration with one of the authors, KT, to the handling Editor. Rie Koide, Kyoto University, Kyoto, contributed to the review of NP and declared a shared affiliation, with no collaboration, with several of the authors, YY and KT, to the handling Editor at the time of review.
