# Supplementary material for: Development of an RNA Virus-Based Episomal Vector Capable of Switching Transgene Expression
Source: Front Microbiol. 2019 Nov 6;10:2485. doi: 10.3389/fmicb.2019.02485 (PMC6851019; doi:10.3389/fmicb.2019.02485)
Supplement: Supplementary file 1 [file Table_1.DOCX]

**Table S1 List of primers used in this study.**

| Primer name | Sequence 5' to 3' | Description |
| --- | --- | --- |
| L2bulge9-front-F | AAACAAACAAAGCTGTCACCGGATGTGCT  TTCCGGTCTGATGAGTCCGTT | L2b9 synthesis |
| L2bulge9-front-R | AAGGGCATCAAGACGATGCTGGTATTGGA  CAACGGACTCATCAGACCGGA | L2b9 synthesis |
| L2bulge9-rear-F | TCGTCTTGATGCCCTTGGCAGTGGATGGG  GACGGAGGACGAAACAGCAAA | L2b9 synthesis |
| L2bulge9-rear-R | TTAATTAAAAAAAAAATTTTTATTTTTCT  TTTTGCTGTTTCGTCCTCCGT | L2b9 synthesis |
| REVec-genome-RT | GTTGCGTTAACAACAAACCAATCAT | RT-PCR for REVec genome RNA |
| REVec-antigenome-RT | TGCGCTACAACAAAGCAACAACC | RT-PCR for REVec antigenome RNA |
| REVec-F | ATGCATTGACCCAACCGGTA | qRT-PCR for REVec genome or antigenome RNA |
| REVec-R | ATCATTCGATAGCTGCTCCCTTC | qRT-PCR for REVec genome or antigenome RNA |
| REVec-probe | FAM-AGAACCCCTCCATGATCTCAGAC  CCAGA-TAMRA | Probe qRT-PCR for REVec |
| GLuc-F | ACCTACGAAGGCGACAAAGA | qRT-PCR for GLuc |
| GLuc-R | TTGTGCAGTCCACACACAGA | qRT-PCR for Gluc |
| Vero-b-actin-F | GCGCGGCTACAGCTTCACCAC | qRT-PCR for Vero β-actin |
| Vero-b-actin-R | GGGCGCCAGGGCAGTAATCTC | qRT-PCR for Vero β-actin |
| REVec-P/M-F | GACCTCCTCTACGCATCAAC | Amplifying REVec P/M region |
| REVec-P/M-R | CATCCAGGGACGATTACCTT | Amplifying REVec P/M region |
