# Supplementary material for: Development of an RNA Virus-Based Episomal Vector Capable of Switching Transgene Expression
Source: Front Microbiol. 2019 Nov 6;10:2485. doi: 10.3389/fmicb.2019.02485 (PMC6851019; doi:10.3389/fmicb.2019.02485)
Supplement: Supplementary file 2 [file Data_Sheet_1.pdf]

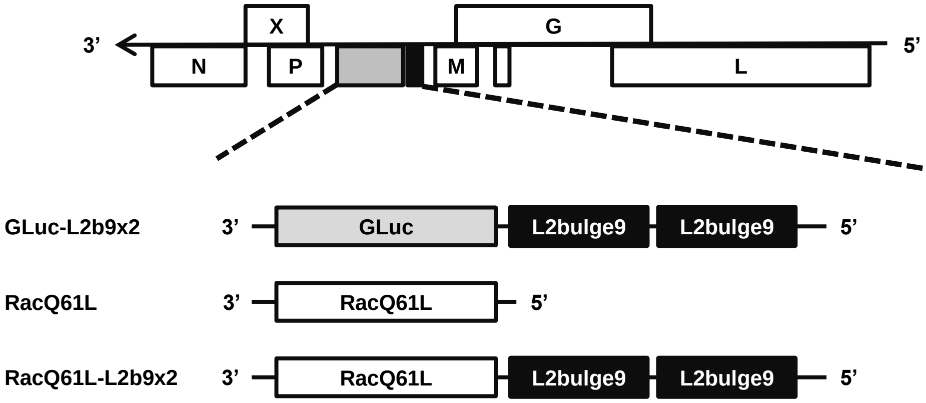

Supplementary Figure S1
Representation of the REVec-Gluc-L2b9x2, REVec-RacQ61L, and REVec-RacQ61L-L2b9x2 vector genomes.

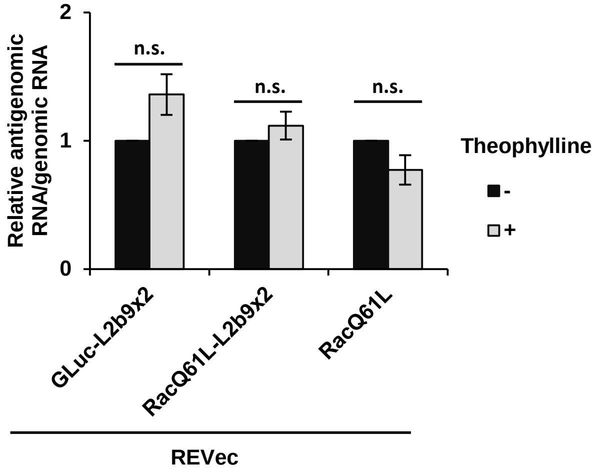

Supplementary Figure S2
Quantification of RNA expression in REVec-GLuc-L2b9x2-, REVec-RacQ61L-L2b9x2- and REVec-RacQ61L-infected Vero cells by qRT-PCR. The amount of REVec antigenomic RNA was measured and standardized against that of REVec genomic RNA. Values are expressed as the mean ± S.E. n.s., no significance (Student's *t* test). At least three experiments were performed.

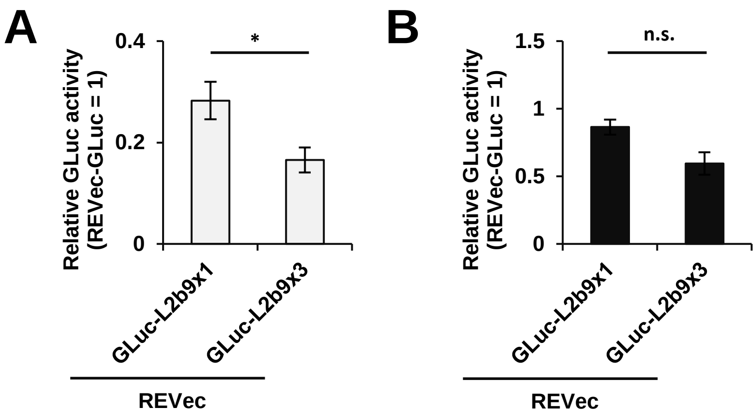

Supplementary Figure S3
Relative luciferase activity after 24 h of treatment with theophylline. Relative luciferase activities from REVec-Gluc-L2b9x1 and REVec-Gluc-L2b9x3 in the presence of 0 (A) and 10 (B) mM theophylline in Figure 2A were shown. Values are expressed as the mean ± S.E. * $P < 0.05$; n.s., no significance (Student's *t* test). At least three experiments were performed.
